# Sequential introduction of single room isolation and hand hygiene campaign in the control of methicillin-resistant *Staphylococcus aureus *in intensive care unit

**DOI:** 10.1186/1471-2334-10-263

**Published:** 2010-09-07

**Authors:** Vincent CC Cheng, Josepha WM Tai, WM Chan, Eric HY Lau, Jasper FW Chan, Kelvin KW To, Iris WS Li, PL Ho, KY Yuen

**Affiliations:** 1 Department of Microbiology, Queen Mary Hospital, Hong Kong Special Administrative Region, China; 2 Infection Control Unit, Queen Mary Hospital, Hong Kong Special Administrative Region, China; 3 Intensive Care Unit, Queen Mary Hospital, Hong Kong Special Administrative Region, China; 4 School of Public Health, The University of Hong Kong, Hong Kong Special Administrative Region, China; 5 Carol Yu Centre for Infection, The University of Hong Kong, Hong Kong Special Administrative Region, China

## Abstract

**Background:**

After renovation of the adult intensive care unit (ICU) with installation of ten single rooms, an enhanced infection control program was conducted to control the spread of methicillin-resistant *Staphylococcus aureus *(MRSA) in our hospital.

**Methods:**

Since the ICU renovation, all patients colonized or infected with MRSA were nursed in single rooms with contact precautions. The incidence of MRSA infection in the ICU was monitored during 3 different phases: the baseline period (phase 1); after ICU renovation (phase 2) and after implementation of a hand hygiene campaign with alcohol-based hand rub (phase 3). Patients infected with extended spectrum beta-lactamase (ESBL)-producing *Escherichia coli *and *Klebsiella species *were chosen as controls because they were managed in open cubicles with standard precautions.

**Results:**

Without a major change in bed occupancy rate, nursing workforce, or the protocol of environmental cleansing throughout the study period, a stepwise reduction in ICU onset nonbacteraemic MRSA infection was observed: from 3.54 (phase 1) to 2.26 (phase 2, p = 0.042) and 1.02 (phase 3, p = 0.006) per 1000-patient-days. ICU onset bacteraemic MRSA infection was significantly reduced from 1.94 (phase 1) to 0.9 (phase 2, p = 0.005) and 0.28 (phase 3, p = 0.021) per 1000-patient-days. Infection due to ESBL-producing organisms did not show a corresponding reduction. The usage density of broad-spectrum antibiotics and fluoroquinolones increased from phase 1 to 3. However a significant trend improvement of ICU onset MRSA infection by segmented regression analysis can only be demonstrated when comparison was made before and after the severe acute respiratory syndrome (SARS) epidemic. This suggests that the deaths of fellow healthcare workers from an occupational acquired infection had an overwhelming effect on their compliance with infection control measures.

**Conclusion:**

Provision of single room isolation facilities and promotion of hand hygiene practice are important. However compliance with infection control measures relies largely on a personal commitment, which may increase when personal safety is threatened.

## Background

Control of nosocomial transmission of methicillin-resistant *Staphylococcus aureus *(MRSA) has been a great challenge to infection control professionals. Intensive care units (ICUs) are often considered as the most important reservoirs for dissemination of MRSA to patients throughout the entire hospital network [1]. Attempts to control the spread of MRSA in ICUs using a series of control measures such as active surveillance cultures, contact isolation of colonized or infected cases, decolonization therapy, and antimicrobial stewardship programs have been met with variable degrees of success [2-6]. However, implementation of these infection control practices is usually limited by shortage of isolation facilities and manpower relative to the large number of colonized or infected patients [6]. Furthermore, the relative importance of the individual measures remains questionable [2,5,7,8].

With the renovation of the ICU in early 2004 as a contingency plan for emerging infectious diseases such as the severe acute respiratory syndrome (SARS) outbreak in 2003, single rooms with either positive or negative pressure were incorporated. We conducted an enhanced infection control program for MRSA in ICU by moving all MRSA colonized or infected patients into single rooms under strict contact precautions while those infected with extended spectrum beta-lactamase (ESBL)-producing organisms were managed in open cubicles under standard precautions as internal controls. In addition, a hand hygiene campaign using alcohol-based hand rub was initiated in ICU in mid 2006. We monitored the occurrence of MRSA infection in ICU during different phases of interventions. However, as the outbreak of SARS which occurred in 2003 may serve as a potential confounder in our quasi-experimental study, we therefore also compared the occurrence of MRSA infection before and after SARS.

## Methods

### Setting of ICU

This study was performed in Queen Mary Hospital, a 1500-bed tertiary referral university-affiliated teaching hospital with a 20-bed ICU managing patients aged 18 years or older from all clinical specialties. Between 1 January 2002 and 31 March 2004, the ICU was located in ward C2 and C4 with a total of 3 single rooms. The single rooms were usually reserved for critically-ill patients with haematological malignancy, transplantation (bone marrow or solid organ), or smear-positive pulmonary tuberculosis. Other beds in open cubicles were separated by at least 3 feet. Renovation of ICU lasted between January 2004 and March 2004. Ten single rooms including 2 with positive pressure and 6 with negative pressure were incorporated in the newly renovated ICU, ward C2E2. A total of 28 washing basins and 63 hangers for alcohol-based hand rub were installed in the single rooms, open cubicles and corridor. The total number of admissions, patient-days, and occupancy rate in ICU were collected from the hospital record office, while data on nursing manpower and the usage density of antibiotic consumption expressed as defined daily dose (DDD) per 1000-patient-days were obtained from the hospital administration office.

### Infection control program for MRSA in ICU

Samples were collected for microbiological investigation when clinically indicated as active surveillance of asymptomatic colonization was not routinely performed in the ICU due to resource limitation. MRSA was identified according to our previous laboratory protocol [9]. New cases of MRSA were identified by the infection control team by checking with the computerized reports of the microbiology laboratory on a daily basis. Patient demographic information, vital signs, use of antibiotics, and the site of MRSA isolation were reviewed at the bedside to determine the route of acquisition and to differentiate colonization from infection. MRSA colonization was defined as asymptomatic isolation of MRSA from clinical samples collected from non-sterile body sites. MRSA infection was defined when the clinical symptoms were correlated with the site of MRSA isolation or when the MRSA was cultured from sterile body sites. Pre-ICU onset MRSA infection was defined as the MRSA infection diagnosed before or within 48 hours of ICU admission, whereas ICU onset MRSA infection referred to the MRSA infection diagnosed after 48 hours of ICU admission. The infection control team also advised healthcare workers in ICU on the isolation and infection control precautions in cases of colonization and infection. Single room isolation with contact precautions including wearing gloves and gown during close patient care was recommended. When the single rooms were fully occupied, cases were managed in open cubicles by cohort nursing. Healthcare workers were reminded to comply with the standard and contact precautions. Routine environment cleansing was performed by soap and water. Disinfection by sodium hypochlorite (1000 ppm) was done upon patient discharge.

A hand hygiene campaign with alcohol-based hand rub was promoted in mid 2006 and fully implemented in ICU since late September 2006 [10]. Four briefing sessions with on-site demonstrations and 3 discussion sessions were held for the frontline staff in ICU. Posters were put up along the corridors and at the room entrances. Skin tolerance to alcohol-based hand rub was assessed by using validated scales to evaluate participants' skin state for redness, scaling, and fissures on a skin scoring scale as previously described [11,12]. Staff were welcomed to consult the infection control team in case of any problems related to skin irritation or damage. Compliance with hand hygiene was regularly audited by infection control nurses who had been trained in the use of a structured observation form and tested for concordance among observers using a pilot of 20 opportunities. All health care workers who provided direct patient care in the ICU were observed randomly and unobtrusively for about twenty minutes when the observers performed surveillance of device-related infection during weekdays. At least 200 hand hygiene opportunities were observed with post-observational feedback provided to the top leaders [13]. The consumption of alcohol-based hand rub in terms of volume used per 1000-patient-days was retrieved from the hospital pharmacy.

### Changes in the incidence density of MRSA and ESBL-producing organisms over time

The incidence density of pre-ICU onset MRSA infection, ICU onset nonbacteraemic MRSA infection, and ICU onset bacteraemic MRSA infection were expressed as number per 1000-patient-days and analyzed according to different phases. Phase 1 was defined as the period before ICU renovation (1 January 2002 to 31 March 2004); phase 2 was defined as the period after ICU renovation (1 April 2004 to 30 June 2006); and phase 3 was defined as the period after implementation of hand hygiene campaign (1 July 2006 to 30 June 2009).

The incidence density of infection due to ESBL-producing *E. coli *and *Klebsiella species *in ICU in different phases were used as controls. Pre-ICU onset infection was defined as the infection diagnosed before or within 48 hours of ICU admission, whereas ICU onset nonbacteraemic or bacteraemic infection due to ESBL-producing organisms referred to those cases with infection diagnosed after 48 hours of ICU admission. In view of the limited number of single rooms even after ICU renovation, patients with infection due to ESBL-producing organisms were not transferred to single rooms for isolation.

Changes in the trend or level of incidence density of ICU onset infection due to MRSA and ESBL-producing organisms from interrupted time-series at different phases as well as before and after SARS were also analyzed.

### Ethical approval

This study was approved by the Institutional Review Board at Queen Mary Hospital

### Statistical Analysis

T-test, Chi-square test, and Fisher's Exact test were used in the analysis where appropriate. Changes in the incidence density of infection due to MRSA and ESBL-producing organisms over time were analyzed by Poisson regression. Trend analysis was performed to evaluate the overall pattern of changes on outcomes of interest over time using interrupted time series with segmented regression analysis. All reported p-values were two-sided. A p-value of <0.05 was considered statistically significant. Computation was performed using R Version 2.8.1.

## Results

### Setting of ICU

**Figure 1:**
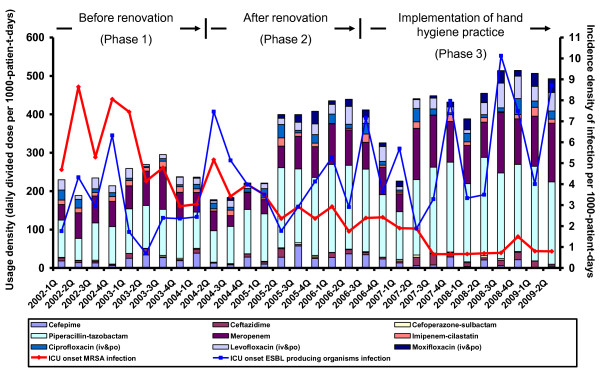
**The trend of usage density of broad-spectrum antibiotics, fluoroquinolones, and incidence density of ICU onset MRSA infection**. Note. ICU, adult intensive care unit; broad-spectrum antibiotics, include cefepime, ceftazidime, and cefoperazone-sulbactam, piperacillin-tazobactam, meropenem, imipenem-cilastatin; fluoroquinolones, include ciprofloxacin, levofloxacin, and moxifloxacin; MRSA; methicillin-resistant *Staphylococcus aureus*. The trend of ICU onset ESBL-producing organisms' infection is listed as reference.

**Figure 2:**
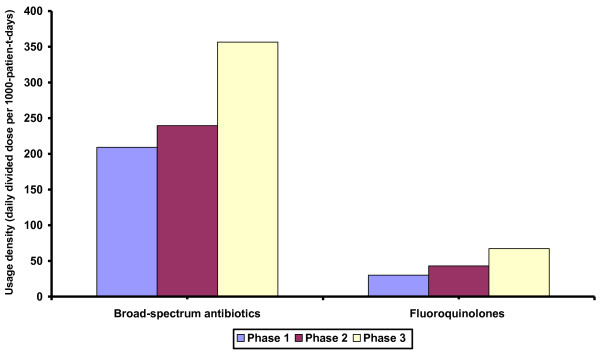
**The overall usage density of broad-spectrum antibiotics & fluoroquinolones in ICU during different phases of interventions**. Note. ICU, adult intensive care unit; broad-spectrum antibiotics, include cefepime, ceftazidime, and cefoperazone-sulbactam, piperacillin-tazobactam, meropenem, imipenem-cilastatin; fluoroquinolones, include ciprofloxacin, levofloxacin, and moxifloxacin.

Between January 2002 and June 2009, there were 12073 patients admitted into ICU with a total of 48167 patient-days, of which 8501 patients (33194 patient-days) were admitted after renovation (Table 1). The occupancy rate in ICU ranged between 83.3% and 97.3% throughout the study period. The nursing manpower was maintained at a ratio of 1 nurse to 1 patient at day time and 1 nurse to 2 patients at night shift. The quarterly consumption patterns of antibiotics were shown in Figure 1, and the increasing trend of overall usage density of broad-spectrum antibiotics (cefepime, ceftazidime, cefoperazone-sulbactam, piperacillin-tazobactam, meropenem, and imipenem-cilastatin), and fluoroquinolones were illustrated in Figure 2.

**Table 1:** Admission data of patient managed in ICU during different phases of interventions.

	Before ICU renovation (1^st ^quarter 2002 to 1^st ^quarter 2004) Phase 1	After ICU renovation (2^nd ^quarter 2004 to 2^nd ^quarter 2006) Phase 2	P value (comparison between phase 1 & 2)	Hand hygiene campaign (3^rd ^quarter 2006 to 2^nd ^quarter 2009) Phase 3	P value (comparison between phase 2 & 3)
Total number of patient-days	14973	15501		17693	
Total number of admission	3572	3477		5024	
Mean number of admission to ICU per quarter (± S.D.)	397 (± 44)	386 (± 53)	0.650	419 (± 27)	0.121

### Infection control program for MRSA in ICU

A total of 553 ICU patients had MRSA cultured from clinical samples and were assessed by the infection control team at the bedside during the study period. Three-hundred and thirty-three (60%) patients had MRSA isolated 48 hours after ICU admission. One-hundred and fifty-four (46%) out of the 333 cases were found to have ICU onset MRSA infection, of which 108 were male and 46 were female. The median age (range) was 68 (19-90) years. The initial sites of positive MRSA cultures were from the lower respiratory tract (82, 53%), bloodstream (48, 31%), skin and soft tissue (13, 8%), intravenous catheter (7, 5%), peritoneum (3, 2%), and urine (1, 1%). In phase 1, 82 patients with ICU onset MRSA infection were managed in open cubicles with cohort nursing. In phases 2 and 3, 49 and 23 MRSA cases were transferred to single rooms and managed under contact precautions respectively. Gloves and gowns were worn by healthcare workers for close patient contact. The protocol of routine daily environmental cleansing remained unchanged throughout the study period.

Unobtrusive hand hygiene observation of at least 200 opportunities per assessment was performed in ICU after the implementation of hand hygiene campaign. The overall compliance rate of hand hygiene increased from 29% (2^nd ^quarter of 2006 as baseline) to 46% (4^th ^quarter of 2006), 54% (4^th ^quarter of 2007), and 64% (3^rd ^quarter of 2008), whereas the compliance of hand hygiene maintained at 63% at the time of writing (3^rd ^quarter of 2009). The consumption of alcohol-based hand rub gradually increased from 24 litres per 1000-patient-days in 2006 to 54.8 litres in 2007, 63.2 litres in 2008, and 167.3 litres per 1000-patient-days in the first 2 quarters of 2009.

### Changes in the incidence density of MRSA and ESBL-producing organisms' occurrence over time

**Figure 3:**
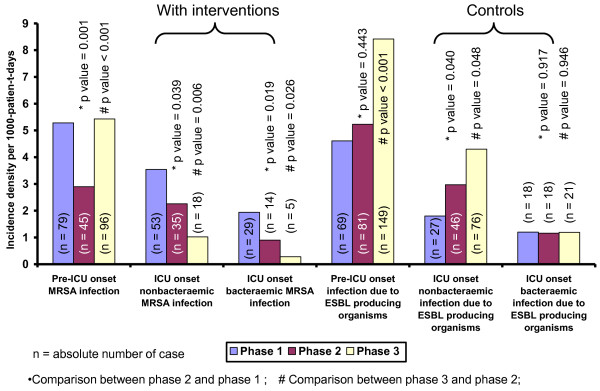
**Changes in the incidence density of infection due to MRSA and ESBL-producing organisms (per 1000-patient-days) in ICU**. Note. ICU, adult intensive care unit; MRSA, methicillin-resistant *Staphylococcus aureus*. ESBL-producing organisms includes *E. coli *and *Klebsiella pneumoniae*.

The incidence density of ICU onset MRSA infection gradually decreased from the peak of 8.65 per 1000-patient-days (second quarter of 2002) to 0.79 per 1000-patient-days (second quarter of 2009) (Figure 1). There was a stepwise and significant reduction in the incidence density of ICU onset nonbacteraemic MRSA infection and ICU onset bacteraemic MRSA infection from phase 1 to 3, but such findings were not observed in the ICU onset infection due to ESBL-producing organisms (Figure 3).

**Figure 4:**
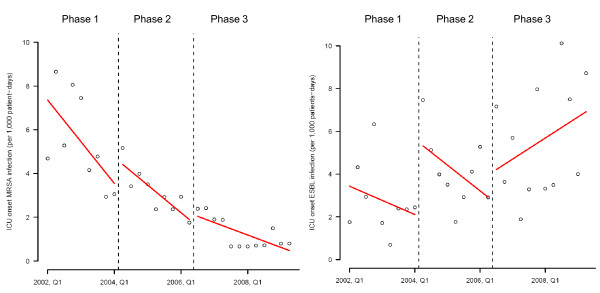
**Change in incidence density of ICU onset infection due to MRSA and ESBL-producing organisms from interrupted time-series with segmented regression analysis according to different phases of interventions**.

**Figure 5:**
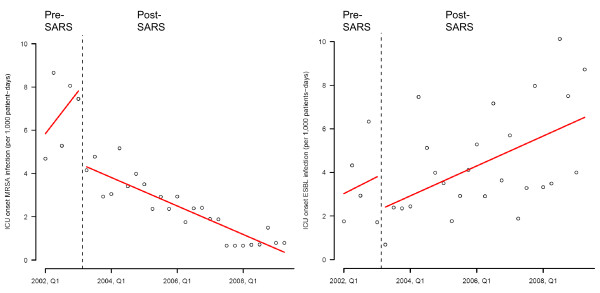
**Change in incidence density of ICU onset infection due to MRSA and ESBL-producing organisms from interrupted time-series with segmented regression analysis before and after the outbreak of severe acute respiratory syndrome (SARS) in 2003 2Q**. Note. Pre-SARS period is defined as the time between 2002 1Q to 2003 1Q and post-SARS period is defined as the time between 2003 2Q to 2009 2Q.

Both the level change and trend change of the incidence density of ICU onset infections due to MRSA and ESBL-producing organisms had no significant difference across different phases during the study period (Table 2, Figure 4). When the incidence density of MRSA infection was analyzed according to the onset of SARS at the second quarter of 2003, the level change (-3.337, p < 0.001) and trend change (-0.658, p = 0.021) of ICU onset MRSA infection, but not infections due to ESBL-producing organisms, were shown to be significantly changed from an increase to decrease (Table 2, Figure 5).

**Table 2:** Change in incidence density of ICU onset infection due to MRSA and ESBL-producing organisms from interrupted time-series with segmented regression analysis during the entire study duration.

Period comparison	ICU MRSA infection	ICU onset infection due to ESBL-producing organisms
Phase 2 vs Phase 1		
Level change	1.188 (p = 0.252)	3.541 (p = 0.091)
Trend change	0.161 (p = 0.415)	-0.135 (p = 0.727)
		
Phase 3 vs Phase 2		
Level change	0.290 (p = 0.757)	1.052 (p = 0.570)
Trend change	0.173 (p = 0.300)	0.547 (p = 0.103)
		
Post-SARS vs Pre-SARS*		
Level change	-3.337 (p < 0.001)	-1.560 (p = 0.413)
Trend change	-0.658 (p = 0.021)	-0.022 (p = 0.975)

Note. SARS, severe acute respiratory syndrome

* Pre-SARS period is defined as the time between 2002 1Q to 2003 1Q and post-SARS period is defined as the time between 2003 2Q to 2009 2Q

## Discussion

We attempted to analyze the sequential effect of single room isolation and promotion of hand hygiene practice using alcohol-based hand rub during our enhanced infection control program, which has achieved a 2 to 3-fold stepwise reduction of ICU onset MRSA infection over a period of 5 years. When all the patients with MRSA colonization or infection were transferred to single rooms with contact isolation, the incidence density of ICU onset MRSA infection were significantly reduced from phase 1 to phase 2 by Poisson regression but not the trend analysis. This was because the incidence density of ICU onset MRSA infection had already decreased in the second half of phase 1 (2003 2Q to 2004 2Q). The outbreak of SARS in Hong Kong in 2003 2Q [14,15] with 8 deaths among healthcare workers [16] greatly enhanced the compliance to infection control measures including handwashing practice among the frontline healthcare workers for a sustained period of time [17]. This change in habit could be a confounder underscoring the effect of single room isolation using interrupted time series analysis. In fact, the incidence density of ICU onset MRSA infection had a significant change in level and trend before and after SARS. This finding illustrates an important principle that the compliance with infection control practices by healthcare workers is determined by the perception of staff that their own personal safety may be threatened if such measures are not followed. The overwhelming effect of SARS on the risk perception by ICU healthcare workers was reported by over 77% of our staff who have participated in the questionnaire survey earlier on reporting a higher rate of hand cleansing practice after exposure to patient's body fluids [10]. The outbreak of SARS may undermine the contribution of using single room isolation in the control of MRSA infection. However, the incidence density of ICU onset infection due to ESBL-producing organisms steadily increased after the outbreak of SARS when patients infected with ESBL-producing organisms were not managed in the single rooms (Figure 5).

The use of single room isolation as the predominant measure to control the spread of MRSA in ICU remains controversial in the literature (Table 3) [2-4,7,8]. A well designed study failed to demonstrate any significant reduction in the incidence of nosocomial acquisition of MRSA in 2 intensive care units where MRSA carriers were managed in single rooms [7]. The low compliance rate with hand hygiene practice of 21% was considered as the major limiting factor to successful infection control. Poor adherence to hand hygiene practice negated the effect of hospital renovation with the provision of single rooms [18]. Therefore, the implementation of hand hygiene has become the crucial factor in the control of MRSA. As shown in our study, despite a significant increase in the incidence density of pre-ICU onset MRSA infection which may increase the colonization pressure in ICU [19], the incidence density of both ICU onset nonbacteraemic and bacteraemic MRSA infection further decreased in phase 3 when hand hygiene campaign was promoted which resulted in a sustained improvement in the compliance rate of over 60% across all ranks of healthcare workers.

**Table 3:** Review of literature on the single room isolation and/or hand hygiene practice as the predominant measures to control the spread of methicillin-resistant *Staphylococcus aureus *in adult intensive care unit.

Study [reference]	Design and setting	Main intervention	Major outcome	Remark
Cepeda JA et al (2005) [7]	Prospective 12-month study in the ICU of 2 teaching hospitals (18-bed for hospital A & 10-bed for hospital B), London, UK	Phase 1 (6-month): all MRSA-positive patients were moved to single room or cohort nursed Phase 2 (7-12 month): all MRSA-positive patients were not moved or cohort nursed Other measures: (i) admission and weekly screening for MRSA colonization; (ii) hand hygiene was encouraged and compliance audited	MRSA acquisition rates in ICU were similar in phase 1 and 2	Suboptimal patient screening, delay in the availability of MRSA results, and low adherence to hand hygiene (21%)
				
Huang SS et al (2006) [2]	Retrospective 9-year study in 8 ICUs in an 800-bed hospital, Boston, US	Phase 1 (since 1 Sept 2000): campaign for sterile CVC placement Phase 2 (since 1 Sept 2001): institution of alcohol-based hand rubs Phase 3 (since 1 Jul 2002): hand hygiene campaign Phase 4 (since 1 Sept 2003): routine admission and weekly screening for MRSA colonization and initiation of contact isolation precaution	Significant reduction in MRSA bacteremia by 75% (p = 0.007) in ICU during phase 4	Other interventions were not associated with a significant change in MRSA bacteremia
				
Bracco D et al (2007) [3]	Prospective 30-month study in a 18-bed medico-surgical ICU (6 single-bed rooms plus a 6-bed and 2-bed bay room), Montreal, Canada	Placement of patients into single room or bay room according to the availability of place Other measures: (i) admission and weekly screening for MRSA colonization; (ii) hand hygiene practice with alcohol-based hand solution	The rate of MRSA acquisition was significantly lower in single room (1.3 per 1000-patient-days) than bay room (4.1 per 1000-patient-days) (p < 0.001)	Placement in single room may reduce MRSA cross-transmission in the institution where MRSA is not hyperendemic
				
Gastmeier P et al (2004) [4]	Questionnaire surveillance to 212 ICUs participating in KISS	To enquire the infection control practice in preventing nosocomial MRSA infection; univariate and multivariate analyses to identify risk factors for nosocomial MRSA infection	164 (77.4%) ICUs response; placement in isolation rooms or cohorts was found to be a protective factor (OR, 0.36; CI_95_, 0.17-0.79) in multivariate analysis	Up to 34% of the German ICUs have not isolated MRSA patients in single rooms or cohorts
				
Harrington G et al (2007) [5]	Prospective 40-month study in a 35-bed ICU, Melbourne, Australia	Introduction of antimicrobial hand hygiene gel with the consumption of hand hygiene product increased from 78.1 liters per 1000-patient-days to 102.7 liters per 1000-patient-days Other measures: MRSA surveillance feedback program using statistical process control chart	The rate of MRSA acquisition was significant lower in post-intervention (6.7 per 100 patient admission) than baseline (9.3 per 100 admission) (p = 0.047)	No admission and weekly screening for MRSA; no placement of MRSA patient in single room
				
Souweine B et al (2009) [8]	Prospective 4-month study in 2 ICUs (10-bed in a University hospital and 8-bed in a non-teaching hospital), France	Provision of alcohol-based hand rub during the intervention period Other measures: (i) admission and discharge screening for MRSA; (ii) decolonization of MRSA patients with mupirocin nasal ointment	No significant reduction in MRSA colonization and infection after intervention	The sample size was underpower to estimate the difference

Note. CI_95_, 95% confidence interval; CVC, central venous catheter; ICU, intensive care unit; KISS, Krankenhaus Infektions Surveillance System (German Nosocomial Infection Surveillance System); MRSA, methicillin-resistant *Staphylococcus aureus*; OR, odd ratio

Whether the use of single room isolation or hand hygiene practice is more important in the control of MRSA in ICU requires further investigation. Previous studies demonstrated that the rate of MRSA acquisition could be significantly reduced by promotion of hand hygiene practice even when single room isolation was not available [5], whereas another report suggested that the rate of MRSA bacteraemia in ICU was increasing during hand hygiene campaign until the initiation of single room isolation of MRSA cases (Table 2) [2]. In our study, we considered that the effect of hand hygiene campaign could be further enhanced when the isolation facilities were in place. The fact that index patients were managed in single rooms with the doors closed served as an important reminder to the attending healthcare workers to perform all necessary infection control practices including hand hygiene according to the five moments recommended by WHO [20]. It could be one of the reasons why the incidence density of infection due to ESBL-producing organisms did not demonstrate a corresponding reduction despite the promotion of hand hygiene practice in ICU in phase 3. In addition, the rise in incidence density of ICU onset infection due to ESBL-producing organisms despite the improved compliance of hand hygiene may be explained by an increase of patients who were already colonized with ESBL-producing organisms upon admission. This reflects a global trend of increasing community carriage of ESBL-producing organisms. However, this point cannot be further elucidated as there was no admission screening performed in this study.

Antibiotic exposure is a well established risk factor for acquisition of MRSA [21]. Our previous study also demonstrated that the microbial load of MRSA in the anterior nares positively correlated with the use of beta-lactam antibiotics and fluoroquinolones [22]. Therefore, we attempted to analyze the use of these antibiotics during the study period. In the absence of antibiotic stewardship program in ICU, the consumption of broad-spectrum antibiotics and fluoroquinolones gradually increased from phase 1 to 3. The result was expected because the incidence of community-acquired infection due to ESBL-producing *E. coli *and *Klebsiella species *increased significantly in our locality [23]. Despite the fact that antibiotic consumption was an important confounding factor for MRSA acquisition [24], we were able to achieve a reasonable control of ICU onset MRSA infection by means of single room isolation and hand hygiene campaign without a significant reduction in antibiotic use.

There are several limitations in this study. Firstly, active surveillance culture was not performed upon ICU admission and during the period of hospitalization in view of resource limitation. The temporal trends of MRSA and ESBL-producing organisms cannot be accurately assessed because the epidemiology of both MRSA and ESBL-producing organisms is currently changing. These hospital associated pathogens are now increasingly found in individuals without risk factors for healthcare exposure [9,23,25]. This may limit the value of using ESBL-producing organisms as a control group. Secondly, molecular typing of the MRSA isolates was not performed among the patients with ICU onset MRSA infection. It is impossible to judge if the interventions reduce the incidence of cross-infection despite a lower rate of MRSA infection observed during the study period. Furthermore, a change in clonal type of MRSA might be a potential confounder for nosocomial transmission. In our previous studies on the molecular epidemiology of blood cultures isolates of MRSA in five hospitals in Hong Kong, including our centre, the MRSA strains related to the CC8/SCC*mec *III/IIIA had decreased from 81.3% before year 2000 to 26.5% in year 2006 to 2008, whereas the CC45/SCCmec IV/V clone had increased from 16.6% to 42.6% in the corresponding period, which was associated with an increasing trend of MRSA bacteremia from 0.05 per 1000 bed-days in 2004 to 0.09 per 1000 bed-days in 2006-2008 [26,27]. It might be one of the reasons why the pre-ICU onset MRSA infection was significantly increased from phase 2 to 3. However, with a combination of single room isolation and enforcement of hand hygiene practice in phase 3, a further reduction of ICU onset MRSA infection was observed despite an increase in colonization pressure in AICU [19].

## Conclusions

ICU onset MRSA infection can be reduced by the provision of isolation facilities and promotion of hand hygiene practice. Moreover, the perception that compliance to infection control practice being an important safeguard of personal safety against infectious disease appears to be an overwhelming factor in the successful implementation of an infection control policy. Further studies should be conducted on how this factor of perception can be exploited by the hospital administration and infection control team in the control of hospital acquired infections and multiple drug resistant organisms.

## Competing interests

The authors declare that they have no competing interests.

## Authors' contributions

VCCC and KYY designed, executed and supervised the study. JWMT collected the clinical information. WMC, JFWC, KKWT, and IWSL participated in the patient management. VCCC and JFWC drafted the manuscript. EHYL conducted the statistical analysis. PLH critically reviewed the manuscript. All authors have read and approved the final manuscript.

## Pre-publication history

The pre-publication history for this paper can be accessed here:

http://www.biomedcentral.com/1471-2334/10/263/prepub
